# Comparison of the value of plasma and urine cystatin-C and neutrophil gelatinase-associated lipocalin levels in prediction of acute kidney injury in sepsis

**DOI:** 10.1186/cc10384

**Published:** 2011-10-27

**Authors:** G Gursel

**Affiliations:** 1 Department of Pulmonary Critical Care Medicine, Gazi University School of Medicine, Ankara, Turkey

## Introduction

The aim was to study the impact of inflammation/sepsis on the concentrations of cystatin-C and neutrophil gelatinase-associated lipocalin (NGAL) in plasma and urine in adult ICU patients and to estimate the predictive properties of cystatin-C and NGAL in plasma and urine for early detection of acute kidney injury (AKI) in patients with sepsis.

## Methods

The RIFLE class for AKI was calculated daily, while plasma and urinary Cys-C and NGAL were determined on days 0 and alternate days until ICU discharge. Test characteristics were calculated to assess the diagnostic performance of urinary and plasma Cys-C and NGAL. The diagnostic and predictive performances of the markers were assessed from the area under the receiver operator characteristic curve (AUC).

## Results

One hundred and twenty-eight patients were studied, and three groups were defined: normal (*n *= 41); sepsis (*n *= 45); and sepsis and AKI (*n *= 42). AUCs for diagnosis of AKI using plasma and uCys-C were as follows: 0.89 (*P *< 0.0001) and 0.91 (*P *< 0.0001) Cut-off points for AKI for plasma and uCys-C were 1.7 mg/l (sensitivity: 83%, specificity: 77%)and 0.11 mg/l (sensitivity = 92%, specificity = 80%), respectively. Urinary NGAL showed fair discrimination for AKI diagnosis (AUC = 0.85). Although plasma NGAL performed less well (AUC = 0.58).

## Conclusion

Plasma and urinary Cys-C are useful markers in predicting AKI in sepsis. pNGAL is raised in patients with sepsis, and should be used with caution as a marker of AKI in ICU patients with sepsis. uNGAL is more useful in predicting AKI as the levels are not elevated in septic patients without AKI.

